# Preoperative chemotherapy in colorectal peritoneal metastatic disease – a real-world observational cohort study

**DOI:** 10.1515/pp-2025-0035

**Published:** 2025-12-11

**Authors:** Peter H. Cashin, Petter Frühling, Pontus Grönlund, Wilhelm Graf, Peter Nygren, Lana Ghanipour

**Affiliations:** Department of Surgery and Urology, 8097 Uppsala University Hospital, Uppsala, Sweden; Department of Surgical Sciences, Uppsala University, Uppsala, Sweden; Department of Oncology, Uppsala University Hospital, Uppsala, Sweden; Department of Immunology, Genetics and Pathology, Uppsala University, Uppsala, Sweden

**Keywords:** colorectal cancer, peritoneal metastases, neoadjuvant therapy, conversion therapy, irinotecan, VEGF-targeted therapy

## Abstract

**Objectives:**

The effects of preoperative chemotherapy have been poorly studied in colorectal cancer with peritoneal metastases (CRC-PM). This study evaluated preoperative chemotherapy from the first multi-disciplinary team meeting (MDT) decision, focusing on the response rates, surgical outcomes, and survival.

**Methods:**

This retrospective cohort study analyzed consecutive patients with resectable or potentially resectable CRC-PM evaluated at Uppsala University Hospital’s peritoneal-MDT between March 2019 and December 2023. Kaplan–Meier curves and Cox-regression analyses were used for survival analysis.

**Results:**

Of 179 patients, 81 underwent upfront surgery; 52 received doublet chemotherapy and 46 received doublet with targeted therapy. Targeted group showed a 52 % overall response rate vs. 36 % for doublet group (p=0.14), with patients selected for CRS and HIPEC at a 52 % vs. 31 % rate, respectively, p=0.086. The median overall survival was superior in the targeted group than in the doublet group (intention-to-treat with all patients included): 21 (95 %CI: 18–35) vs. 17 (95 %CI: 14–22) months (p=0.027). The VEGF-targeted therapy outperformed EGFR-targeted therapy: 32 (95 %CI: 21-Not reached) vs. 15 (95 %CI: 11–40) months (p=0.042).

**Conclusions:**

Preoperative chemotherapy with targeted antibodies improves overall survival in CRC-PM in patients that are not candidates for upfront CRS and HIPEC. Bevacizumab is associated with improvement over EGFR targeted treatment in a subgroup analysis.

## Introduction

Colorectal cancer (CRC) remains a significant global health challenge, as it is the third most prevalent cancer worldwide and a frequent cause of cancer-related deaths [1]. Peritoneal metastases (PM) represent a particularly aggressive form of metastatic CRC with historically poor outcomes [2], 3]. The introduction of cytoreductive surgery (CRS) combined with hyperthermic intraperitoneal chemotherapy (HIPEC) has substantially improved the survival of selected patients [4], [5], [6].

Neoadjuvant systemic chemotherapy primarily aims to target micrometastases and thus, improve long-term surgical outcomes. However, the evidence regarding its efficacy in CRC-PM remains inconclusive [7], [8], [9]. FOLFOX and FOLFIRI are the main backbone therapies used to treat metastatic colorectal cancer. Moreover, the addition of VEGF or EGFR antibodies and/or triplet regimens such as FOLFIRINOX may improve the outcome of metastatic disease, with higher rates of conversion to surgery [10]. Interestingly, the VEGF-targeted therapy bevacizumab may have an advantage in peritoneal metastatic setting [11], 12]. These studies are the rationale behind adding neoadjuvant bevacizumab in the ongoing CAIRO 6 trial and were found to be safe prior to CRS+HIPEC [13], 14], even though retrospective studies indicated increased postoperative morbidity after CRS+HIPEC from preoperative bevacizumab [15].

Previous studies have often included patients at the date of cytoreductive surgery, excluding those not selected for surgery, which limits clinically relevant comparisons [7], [8], [9, 11], 12]. In the present study, we used the date of the multidisciplinary therapy conference (MDT) as the starting point, which allowed for an intention-to-treat analysis and enabled us to capture all patients undergoing preoperative chemotherapy for CRC-PM. In Sweden, upfront CRS remains the standard of care, which means that referrals come prior to decision-making on preoperative chemotherapy.

This study aimed to evaluate the efficacy of preoperative systemic chemotherapy in resectable or potentially resectable CRC-PM (excluding the dMMR subtype), focusing on response rates, conversion to surgery, and survival. By providing real-world data, this study could guide the choice of optimal preoperative systemic therapy from available standard regimens.

## Methods

This study was a retrospective, descriptive, real-world observational cohort analysis with subgroup comparisons conducted at Uppsala University Hospital. The cohort comprised of all consecutive patients with CRC-PM evaluated at the HIPEC MDT from March 2019 to December 2023. Data were sourced from single-center hospital digital charting during one-time data retrieval in 2024. Core variables were registered: patient characteristics, tumor characteristics, previous surgical treatment for primary tumor as well as previous anti-tumoral treatment, all anti-tumoral treatments (surgery, radiation, systemic therapy) from the date of 1st MDT evaluation until progression, and date of death. Exposure was considered as the first-choice treatment after 1st MDT evaluation (upfront surgery, doublet chemotherapy, or chemotherapy with targeted agents). The outcomes were response rates, planned surgery, successful CRS, and survival. Patients who met the following criteria were included (Table 1):

1. Inclusion:
  – Colorectal cancer origin.
  – Patients were referred to the peritoneal MDT conference with radiological signs of PM or an intraoperative diagnosis of peritoneal metastases.
  – Synchronous and metachronous PM disease were eligible.
  – Age 18–79.
  – In addition to PM, potentially resectable extra-peritoneal disease defined as limited retroperitoneal lymph node disease, resectable or potentially resectable liver metastases, and resectable or potentially resectable pulmonary metastases were allowed.
2. Exclusion:
  – Patients unable to tolerate at least doublet systemic chemotherapy.
  – Non-curative metastatic disease in addition to PM.
  – dMMR subtype.
  – Appendiceal origin.

**Table 1:** Patient demographics and tumor characteristics.

	Overall	Upfront	Doublet	Targeted	p-Value
	n=179	n=81	n=52	n=46	Doublet vs. targeted
Sex–female	82 (44 %)	40 (49 %)	**19 (36.5** **%)**	**20 (43.5 %)**	0.53
Age mean (+/−SD)	64 ± 12	65 ± 11	**65** ± **10**	**61** ± **13**	0.075
Rectum primary	20 (11 %)	5 (6 %)	**10 (19** **%)**	**5 (11** **%)**	0.40
Colon primary	165 (89 %)	76 (94 %)	**42 (81** **%)**	**41 (89** **%)**	
Positive retroperitoneal lymph-nodes	13 (7 %)	6 (7 %)	**5 (10** **%)**	**2 (4** **%)**	0.98
Liver metastastes	37 (20 %)	10 (12 %)	**14 (27** **%)**	**12 (26** **%)**	0.93
Number of metastases–median [IQR]	2 [1–4]	1 [1-1]	**3 [1–6]**	**2 [2–6]**	0.76
Pulmonary metastases	9 (5 %)	3 (4 %)	**4 (8** **%)**	**1 (2** **%)**	0.22
Synchronous peritoneal metastases	107 (58 %)	42 (52 %)	**31 (60** **%)**	**29 (63** **%)**	0.73
Metachronous peritoneal metastases	78 (42 %)	39 (48 %)	**21 (40** **%)**	**17 (37** **%)**	
Neoadjuvant therapy for primary	16 (9 %)	4 (5 %)	**4 (8** **%)**	**7 (15** **%)**	0.24
Adjuvant therapy for primary	60 (32 %)	31 (38 %)	**14 (27** **%)**	**14 (30** **%)**	0.70
Not selected for surgery	55 (30 %)	0 (0 %)	**31 (59 %)**	**20 (43 %)**	0.086
Cytoreductive surgery	110 (59 %)	69 (85 %)	**16 (31 %)**	**24 (52 %)**	
Open close surgery	20 (11 %)	12 (15 %)	**5 (10 %)**	**2 (4 %)**	
HIPEC					0.37
Not selected for Surgery/HIPEC	55 (30 %)	0 (0 %)	**31 (59** **%)**	**20 (43** **%)**	
Oxaliplatin + IV 5FU	62 (34 %)	42 (52 %)	**7 (14** **%)**	**13 (28** **%)**	
Irinotecan + IV 5FU	22 (12 %)	12 (15 %)	**4 (8** **%)**	**5 (11** **%)**	
Oxaliplatin + irinotecan + IV 5FU	15 (8 %)	9 (11 %)	**3 (6** **%)**	**3 (7** **%)**	
Mitomycin	1 (0.5 %)	1 (1 %)	**0 (0** **%)**	**0 (0** **%)**	
Cisplatin	1 (0.5 %)	1 (1 %)	**0 (0** **%)**	**0 (0** **%)**	
HIPEC not given, but CRS performed	9 (5 %)	4 (5 %)	**2 (4** **%)**	**3 (7** **%)**	
HIPEC not given, open/close surgery	20 (11 %)	12 (15 %)	**5 (10** **%)**	**2 (4** **%)**	
(y)pT2	2 (1 %)	0 (0 %)	**1 (2** **%)**	**1 (2** **%)**	0.84
(y)pT3	56 (30 %)	20 (25 %)	**19 (37** **%)**	**15 (33** **%)**	
(y)pT4	109 (59 %)	56 (69 %)	**24 (46** **%)**	**25 (54** **%)**	
T stage missing	18 (10 %)	5 (6 %)	**8 (15** **%)**	**5 (11** **%)**	
N0	33 (18 %)	18 (22 %)	**7 (13 %)**	**8 (17 %)**	0.70
N1	64 (35 %)	28 (35 %)	**16 (31 %)**	**15 (33 %)**	
N2	67 (36 %)	29 (36 %)	**19 (37 %)**	**18 (39 %)**	
N Missing	21 (11 %)	6 (7 %)	**10 (19 %)**	**5 (11 %)**	
CC score 0	107 (58 %)	68 (84 %)	**15 (29** **%)**	**23 (50** **%)**	0.29
CC Score 1	3 (2 %)	1 (1 %)	**1 (2** **%)**	**1 (2** **%)**	
Open/close surgery	20 (11 %)	12 (15 %)	**5 (10** **%)**	**2 (4** **%)**	
PCI at surgery (mean±SD)^a^	11 ± 9	12 ± 8	**10** ± **11**	**10** ± **10**	0.99
KRAS wildtyp	68 (38 %)	32 (39 %)	**11 (21** **%)**	**25 (54** **%)**	0.0014
KRAS mutated	50 (28 %)	22 (27 %)	**19 (37** **%)**	**9 (20** **%)**	
KRAS missing	61 (34 %)	27 (34 %)	**22 (42** **%)**	**12 (26** **%)**	
BRAF wildtyp	95 (53 %)	45 (55 %)	**25 (48** **%)**	**25 (54** **%)**	0.074
BRAF mutated	21 (12 %)	9 (11 %)	**4 (8** **%)**	**8 (17** **%)**	
BRAF missing	63 (35 %)	27 (34 %)	**23 (44** **%)**	**13 (28** **%)**	
PIK3CA wildtyp	85 (47 %)	46 (57 %)	**16 (31** **%)**	**23 (50** **%)**	0.13
PIK3CA mutated	13 (7 %)	6 (7 %)	**5 (10** **%)**	**2 (4** **%)**	
PIK3CA missing	81 (46 %)	29 (36 %)	**31 (60** **%)**	**21 (46** **%)**	

^a^ Calculated only on the patients selected for surgery. SD, standard deviation; IQR, interquartile range; CC, completeness of cytoreduction; HIPEC, hyperthermic intraperitoneal chemotherapy; PCI, peritoneal cancer index; CRS, cytoreductive surgery; IV, intravenous; 5-FU, 5-fluorouracil. Bold values signify the two preoperative chemotherapy groups being compared.

### Indication for systemic chemotherapy and group allotment

All the patients were referred before treatment initiation for PM. As upfront surgery is the standard of care, the following indications for preoperative systemic chemotherapy were identified (Table 2).

1. Computed tomography scored a peritoneal cancer index (CT-PCI) ≥ 15. If there is a high CT-PCI burden, this indication is identified as the primary cause of preoperative chemotherapy. This group was further divided into extra-peritoneal diseases or isolated PM.
2. For patients with CT-PCI <15, systemic chemotherapy was indicated for:
  – Synchronous primary with indication for systemic therapy (cT4/N2 and rectal cancer)
  – Complex abdominal wall metastases–reduction in size would simplify abdominal wall reconstruction.
  – Complex peritoneal metastasis – reduction in size would simplify resection (e.g., duodenal wall involvement).
  – Extra-peritoneal disease

**Table 2:** Indications, treatment specifics and response rates in the preoperative chemotherapy groups.

	Doublet	Targeted	p-Value	Ox-based	Irino-based	p-Value	EGFR	VEGF	p-Value
	n=52	n=46		n=52	n=34		n=23	n=10	
Peritoneal disease extent			0.051			1.00			0.34
Low CT-PCI <15	38 (73 %)	25 (54 %)		34 (65 %)	22 (65 %)		11 (49 %)	6 (60 %)	
High CT-PCI 15+	13 (25 %)	20 (44 %)		17 (33 %)	11 (32 %)		12 (51 %)	3 (30 %)	
Missing	1 (2 %)	1 (2 %)		1 (2 %)	1 (3 %)		0 (0 %)	1 (10 %)	
Indication for preop chemo			0.52			0.85			0.40
CT-PCI <15, cT4/N2/Rectum	9 (17 %)	6 (13 %)		9 (17 %)	4 (12 %)		2 (9 %)	2 (20 %)	
CT-PCI <15, abdominal wall	2 (4 %)	0 (0 %)		2 (4 %)	0 (0 %)		0 (0 %)	0 (0 %)	
CT-PCI <15, complex periton	5 (10 %)	4 (9 %)		4 (8 %)	4 (12 %)		3 (13 %)	0 (0 %)	
CT-PCI <15, extra-peritoneal	22 (42 %)	15 (33 %)		19 (37 %)	14 (41 %)		6 (26 %)	4 (40 %)	
CT-PCI 15+ *w*/o extra-peritoneal	11 (21 %)	17 (37 %)		14 (27 %)	10 (29 %)		9 (39 %)	3 (30 %)	
CT-PCI 15+ *w*/extra-peritoneal	2 (4 %)	3 (7 %)		3 (6 %)	1 (3 %)		3 (13 %)	0 (0 %)	
Other	1 (2 %)	1 (2 %)		1 (2 %)	1 (3 %)		0 (0 %)	1 (10 %)	
Previous chemo for primary			0.25			0.0036			0.83
None	35 (67 %)	27 (59 %)		42 (81 %)	14 (41 %)		11 (48 %)	6 (60 %)	
Previous ox	8 (15 %)	14 (30 %)		6 (12 %)	13 (38 %)		9 (39 %)	4 (40 %)	
Previous iri	1 (2 %)	1 (2 %)		0 (0 %)	0 (0 %)		1 (4 %)	0 (0 %)	
5-FU only	7 (13 %)	2 (4 %)		4 (8 %)	5 (15 %)		1 (4 %)	0 (0 %)	
Missing	1 (2 %)	2 (4 %)		0 (0 %)	2 (6 %)		1 (4 %)	0 (0 %)	
Preoperative chemo for PM			0.00021			NA			0.72
Ox-based	36 (78 %)	16 (35 %)		52 (100 %)	0 (0 %)		9 (39 %)	4 (40 %)	
Iri-based (incl FOLFOXIRI)	10 (22 %)	24 (52 %)		0 (0 %)	34 (100 %)		11 (48 %)	6 (60 %)	
5-FU only	3 (6 %)	0 (0 %)		–	–		0 (0 %)	0 (0 %)	
Encorafenib	0 (%)	3 (7 %)		–	–		3 (13 %)	0 (0 %)	
Missing	3 (6 %)	3 (7 %)		–	–		0 (0 %)	0 (0 %)	

**Outcomes–response rates**	**Doublet**	**Targeted**	**p-Value**	**Ox-based**	**Irino-based**	**p-Value**	**EGFR**	**VEGF**	**p-Value**

ORR overall	18/50 36 %	23/45 52 %	0.14	21/51 41 %	14/34 41 %	1.0	9/23 39 %	5/10 50 %	0.56
ORR peritoneum	15/48 31 %	21/45 47 %	0.13	19/49 39 %	13/34 38 %	0.96	7/23 30 %	5/10 50 %	0.28
ORR lymph-node	4/8 50 %	2/6 33 %	0.82	4/9 44 %	1/4 25 %	0.51	1/4 25 %	0/0 NA	NA
ORR liver	4/13 31 %	10/11 91 %	0.022	6/10 60 %	4/10 40 %	0.37	4/5 80 %	2/3 67 %	0.67
ORR lung	1/3 33 %	0/1 0 %	0.93	1/2 50 %	0/2 0 %	0.25	0/1 0 %	0/0 NA	NA
Planned surgery			0.086			0.83			0.32
No surgery planned	31 (60 %)	20 (43 %)		28 (54 %)	16 (47 %)		13 (57 %)	4 (40 %)	
Successful cytoreduction	16 (31 %)	24 (52 %)		20 (38 %)	15 (44 %)		8 (35 %)	6 (60 %)	
Open-close surgery	5 (10 %)	2 (4 %)		4 (8 %)	3 (9 %)		2 (9 %)	0 (0 %)	

**Outcomes –** **OS (95 % CI)**	**Doublet**	**Targeted**		**Ox-based**	**Irino-based**		**EGFR**	**VEGF**	

Univariate HR	Ref	0.62 (0.36–1.08)^b^		Ref	0.60 (0.33–1.09)^b^		Ref	0.39 (0.11–1.40)	
Multivariable HR^a^	Ref	0.52 (0.27–1.02)^b^		Ref	0.50 (0.22–1.16)^b^		Ref	0.12 (0.02–1.13)^b^	
Median OS – all patients	17 (14–22)	21 (18–35)		16 (14–22)	26 (18–40)		15 (11–40)	32 (21-NR)	
Median OS – successful CRS	34 (21-NR)	40 (33-NR)		21 (18–40)	NR		NR	NR	

^a^Adjusted for previous chemo, indication for neo, N-stage, KRAS, and BRAF, ^b^p-value ≤0.1. Neo, neoadjuvant; NeoConv, neoadjuvant conversion; Ox, oxaliplatin; Irino, irinotecan; EGFR, epidermal growth factor receptor; VEGF, vascular endothelial growth factor; CT-PCI, computer tomography peritoneal cancer index; PM, peritoneal metastases; ORR, objective response rate; HR, hazard ratio; OS, overall survival, NA, not applicable; NR, not reached, complex periton – indicates a peritoneal metastases with resectibility issues, e.g. duodenal involvement, ureteral involvement, etc.

Although the exact determination of resectability was not always clearly delineated, patients were stratified for assessment of outcome into three groups based on the actual systemic chemotherapy administered:

– Group 1 (Upfront CRS-HIPEC): Direct surgery without neoadjuvant therapy – standard of care
– Group 2 (Doublet Group): Potentially resectable disease given oxaliplatin or irinotecan-based doublet therapy with 5-flourouracil or capecitabine chemotherapy with re-staging at a subsequent MDT prior to the decision to move forward with CRS+HIPEC.
– Group 3 (Targeted Group): Potentially resectable disease where patients received EGFR or VEGF antibody added to the chemotherapy doublet or FOLFOXIRI triplet chemotherapy with re-staging at a subsequent MDT prior to the decision to move forward with CRS+HIPEC.

During chemotherapy, re-staging to evaluate tumor response and deciding on CRS+HIPEC was performed every 2nd month. All patients were evaluated using thoracoabdominal computer tomographic scan at baseline and re-staging. Positron emission tomography was used to evaluate extra-peritoneal diseases. Magnetic resonance tomography (MRI) was used to evaluate all liver metastases. Patients progressing but responding to second-line treatment were included in their baseline treatment group. Survival was calculated from the date of the first MDT decision, regardless of whether it meant upfront CRS-HIPEC or the commencement of systemic therapy, until death from any cause. Data on demographics, previous chemotherapy treatment (if any) related to the primary tumor, response rates, surgical outcomes, and survival outcomes were collected. RECiST 1.1 criteria for response rates were used. To simplify the flowchart, patients with an objective response defined as complete or regression on the evaluation were defined as responders, while patients with mixed or stable or progressive disease were labelled as non-responders. This study was approved by the Ethical Review Authority (Dnr 2023-03743-01 and updated 2023-07012-02). Study was conducted in accordance with the Declaration of Helsinki (updated 2013).

#### Statistics

All consecutive patients were included to avoid selection bias. The flowchart shows the clinical pathway from the first evaluation to successful CRS. Only comparisons between the upfront, doublet, and targeted groups were preplanned. The remaining subgroup analyses were ad hoc: irinotecan vs. oxaliplatin and VEGF vs. EGFR-targeted therapy. Descriptive statistics were used to compare different subgroups. Pearson’s Chi-square statistic, Student’s *t*-test statistic, and Mann-Whitney *U* test were used as appropriate. Kaplan–Meier curves were generated, and Cox’s f-test or log-rank test was used to compare differences in overall survival. Univariate and multivariate Cox regression analyses were used to evaluate hazard ratios and 95 % confidence intervals. Multivariable regression was performed on the systemic treatment groups and subgroup comparisons to adjust for potential confounders defined *a priori* as follows: previous chemotherapy for the primary cancer, indication for systemic chemotherapy, N stage, KRAS, and BRAF. Due to smaller sample sizes overall, differences with p-value ≤0.1 were considered significant if the effect size difference was clinically relevant. All analyses were performed using Statistica v14.

## Results

**Figure 1:**
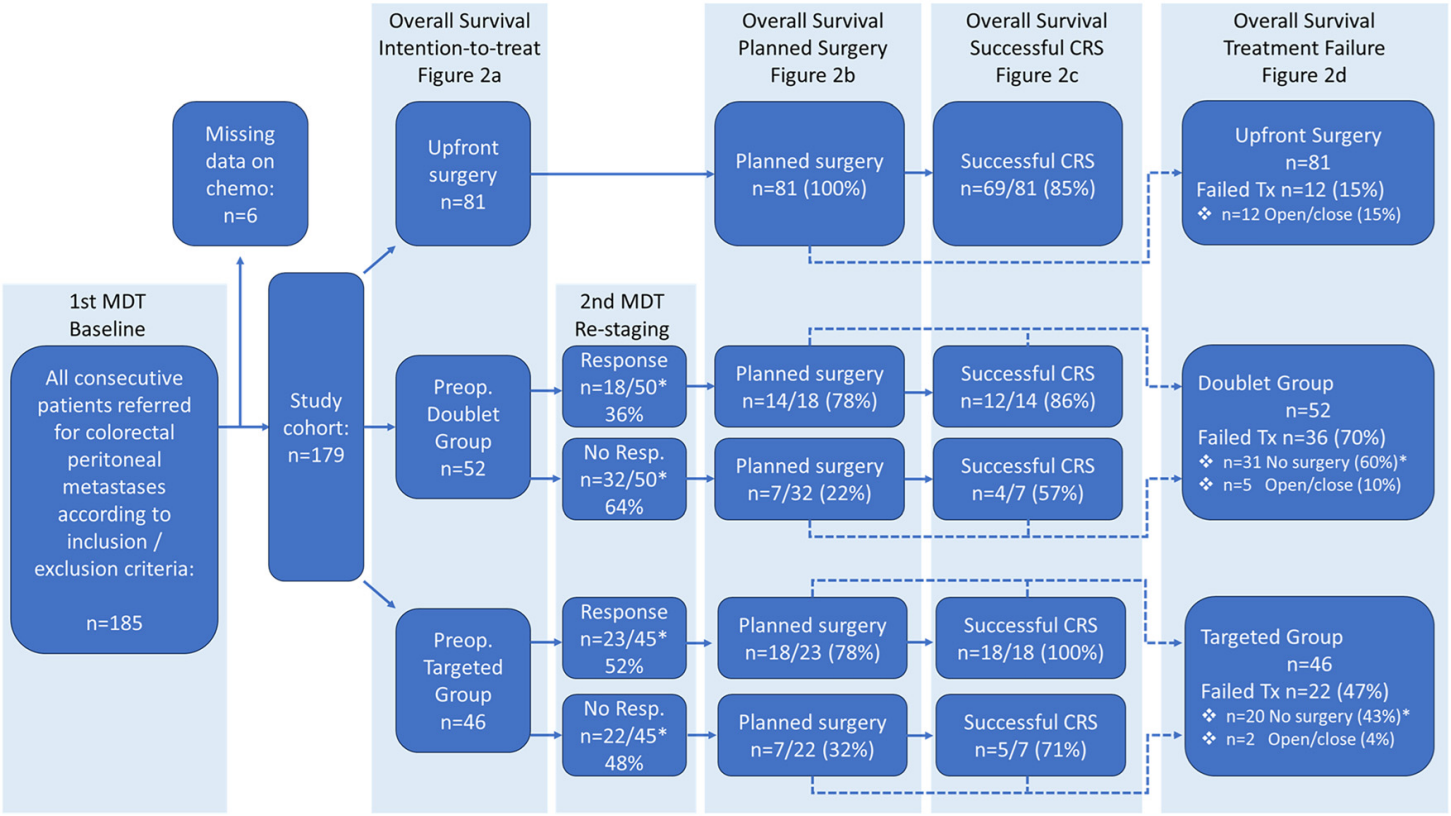
Clinical pathways from MDT decision to successful CRS. *3 patients were missing from response (2 in the doublet group and 1 in the targeted group) due to no visible lesions on CT both at baseline and re-staging but confirmed by previous pathology.

A total of 179 patients were included in the study, as shown in Figure 1. The mean age was 64 (±12) years, with a slight predominance of males (56 %). Table 1 summarizes the demographic and clinical data for each treatment group, with a p-value comparison between groups 2 (doublet group) and 3 (targeted group). Since upfront CRS and HIPEC are still the standard of care in Sweden, the specific indications for systemic treatment were recorded for each patient starting such treatment. Information concerning the treatments received and indications are presented in Table 2.

### Outcomes – response rates and survival

The objective response rates (ORR) for each site of metastasis are provided in Table 2, along with the overall ORR. The number of responses with the corresponding percentage rate is provided. It is noteworthy that targeted therapy had a significant impact on liver metastases, while pulmonary and retroperitoneal lymph node metastases were too few for any relevant comparison.

**Figure 2:**
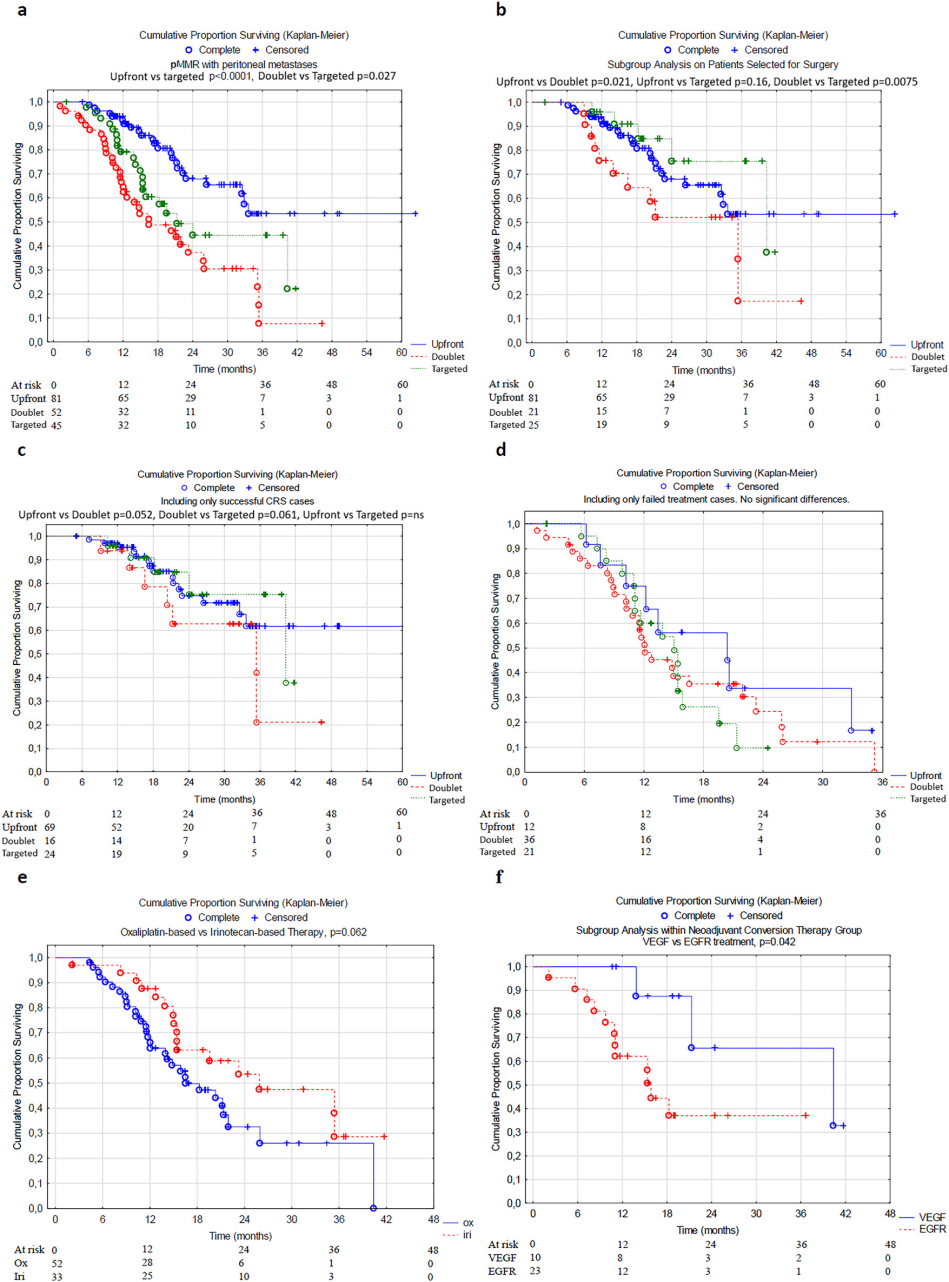
Overall survival curves (a) whole cohort (b) selected for surgical exploration (c) successful CRS (d) failed treatment (i.e. not selected for surgery or unsuccessful surgery) (e) oxaliplatin vs. irinotecan-based preoperative systemic chemotherapy (f) VEGF vs. EGFR-based antibody treatment in the targeted group. ^a^Calculated only on the patients selected for surgery. SD, standard deviation; IQR, interquartile range; CC, completeness of cytoreduction; HIPEC, hyperthermic intraperitoneal chemotherapy; PCI, peritoneal cancer index; CRS, cytoreductive surgery; IV, intravenous; 5-FU, 5-fluorouracil. *These patients switched to irinotecan-based treatment with or without antibodies; ˆone patient switched to cetuximab with encorafenib, and one continued with oxaliplatin-based treatment.

Survival curves are shown in Figure 2. The median overall survival (OS) with 95 % confidence intervals for the doublet and targeted therapy groups are reported in Table 2, along with subgroup analyses. The median OS in the upfront surgery group was not reached (Figure 2a).

In Figure 2, overall survival (OS) is reported depending on each step in the movement towards successful CRS: first, OS was evaluated in all patients entering the study at the first MDT (2a); second, evaluation of OS for those patients selected for surgical exploration (2b); third, evaluation of OS for those undergoing successful CRS (2c); and lastly, evaluation of OS in failed cases (2d). The targeted group performed better in all three stages of the treatment compared to the doublet group (2a-c), while there was no difference in the failed cases (2d). The last two OS comparisons were two subgroup comparisons of oxaliplatin vs. irinotecan-based preoperative chemotherapy (2e) and VEGF vs. EGFR-targeted treatment in the targeted group (2f). Irinotecan-based and VEGF-targeted treatments showed improvements over their counterparts.

## Discussion

The main finding of this study was the superior outcome of targeted therapy over doublet therapy without targeted agents in terms of overall survival. Although statistical significance was not reached for all secondary evaluated outcomes, the numerical trends corroborated the overall survival benefits of this approach. Furthermore, bevacizumab provided benefits over EGFR-targeted therapy in terms of survival. Comparison of irinotecan-based treatment vs. oxaliplatin-based treatment suggested an avenue of further research where irinotecan may have a relevant benefit to evaluate in future studies in this setting.

The data in our study support the use of intensified targeted therapy for preoperative chemotherapy in CRC-PM. Both univariate and multivariate analyses highlighted a clinically relevant improvement with irinotecan; however, this benefit was not evident in the response rates. Although response rates are not always predictive of overall survival, they remain critical markers of treatment efficacy. The significant positive impact of targeting VEGF on the hazard ratio and Kaplan–Meier survival outcomes was somewhat unexpected. Due to the small sample size, a meaningful response rate comparison was not feasible; however, the overall and peritoneal objective response rates improved in the VEGF group compared to the EGFR group (Table 2).

According to recent research, patients with CRC-PM are classified as consensus molecular subtype (CMS) 4 in 75–85 % [16], 17]. A recent review of systemic chemotherapy trials in generalized metastatic CRC suggests that irinotecan may confer a potential advantage in the CMS 4 subtype; in fact, this was observed across the board in the systematic review [18]. To our knowledge, this is the first clinical study on CRC-PM, possibly corroborating this effect with irinotecan treatment. In addition, bevacizumab was also shown to have an effect on CMS 4 in the same systematic review [18]. The relatively hypoxic nature of the peritoneal microenvironment compared with other metastatic sites may be a pathophysiological mechanism that depends on angiogenesis via the VEGF pathway, which is more evident in PM and in the CMS 4 subtype [19]. Bevacizumab treatment may have an advantage in this respect with increased anti-tumoral effects in these patients, which has been suggested not only in our study but also in a few others as well [10], 11].

Owing to the limited number of cases, it was not feasible to perform a subgroup analysis comparing bevacizumab with EGFR-targeted treatments exclusively in patients with wild-type KRAS. It is important to keep this in mind when interpreting the results of our study. Nonetheless, considering peritoneal microenvironment factors and several clinical studies corroborating efficacy, bevacizumab may be a better choice over EGFR-targeted treatment options for peritoneal metastatic disease or CMS 4 subtype.

One of the primary reasons for the better outcome of the targeted group compared with the doublet group was the higher rate of successful CRS. Specifically, the risk of an open/closed surgical result was approximately 15 % in the upfront surgery group, which decreased to 10 % in the doublet group and further to 4 % in the targeted group. Notably, although the targeted group presented with more advanced disease at baseline (44 % high CT-PCI vs. 25 % high CT-PCI, p=0.051), the conversion rate to surgery was higher in the targeted group compared to the doublet group. Moreover, the overall survival of patients with successful CRS in the targeted group was better than that in the doublet group and at least comparable to that of patients in the upfront surgery group (Figure 2).

Our study also highlights the importance of how study cohorts are defined. A retrospective study identifying patients planned for surgery will miss patients failing preoperative chemotherapy, which has been a problem with the previous preoperative treatment studies published [7], [8], [9]. Patients included in a study at the time of preoperative chemotherapy (including borderline resectable patients) will have different intention-to-treat outcomes than those in the planned surgery group. The indication for preoperative therapy is also critical to keep in mind, as this will also determine the success rate of both preoperative chemotherapy and subsequent surgical attempts.

In our study, some patients in the non-responsive group underwent surgery (Table 3). Attempting surgical exploration without further disease control with more chemotherapy appears to lower the chance of successful CRS to 50 %. However, if further disease control with chemotherapy is achieved, the success rate of a surgical attempt increases to 75–100 % (Table 3). It seems reasonable to continue giving these patients the possibility of surgery by allowing further re-evaluations at subsequent MDTs.

**Table 3:** Continued treatment of non-responders after first MDT evaluation of preoperative chemotherapy.

	Doublet	Targeted
n=	32	22
Palliative–no more evaluations at MDT	19 (59 %)	10 (45 %)
Surgical attempt without more chemotherapy	4 (13 %)	4 (18 %)
Successful CRS	2	2
Further chemotherapy with new re-staging	9 (28 %)	8 (36 %)
Going for surgical attempt after 2nd re-staging	2	4
Successful CRS	2^b^	3^a^

^**a**^These patients switched to irinotecan-based treatment with or without antibodies; ^b^one patient switched to cetuximab with encorafenib, and one continued with oxaliplatin-based treatment.

### Limitations

The key limitations of this study include its retrospective design, which inherently introduces selection bias, and the limited cohort size, which reduces statistical power. Patient selection remains a critical aspect of treatment in this setting. In Sweden, upfront CRS+HIPEC remains the standard of care, although preoperative therapy is gaining traction. This differs from practices in other countries, where preoperative chemotherapy is the standard starting point for all patients. Consequently, our findings are most applicable to patients managed under Swedish guidelines where upfront surgery is standard-of-care, and preoperative chemotherapy treatment is recommended for an advanced primary tumor or in a borderline resectable situation, either as high radiological PCI or due to specific metastases that create surgical resectability issues (e.g. duodenal involvement or major abdominal wall involvement, etc.).

While the use of systemic therapy vs. upfront CRS and HIPEC reflects a more homogeneous decision-making process in our cohort, the criteria distinguishing the need for targeted vs. doublet chemotherapy are poorly defined. This ambiguity is evident in Table 2, in which patients, regardless of their initial indication for preoperative chemotherapy, were found in both the doublet and targeted groups. However, when stratifying patients based on high vs. low CT-PCI, it becomes apparent that targeted therapy was more frequently administered to those with high-volume peritoneal disease for conversion reasons. Therefore, the treatment approach for high-volume peritoneal disease may be applicable across most peritoneal surface oncology centers. Despite an increased baseline peritoneal burden, patients selected to undergo surgery had the same surgical PCI level between the doublet and targeted groups. PCI levels in both groups were numerically lower than those in the upfront surgery group, which was unexpected. Since the time to start chemotherapy is generally shorter than the time to surgery, the chemotherapy groups may gain from a suppressed tumor cell turnover rate, which is beneficial during the waiting time from the last chemotherapy cycle to surgery. Conversely, in the upfront group, there may be an increased risk of progression from MDT to surgery because the patient is untreated, even though the waiting time is the same between the different groups, which could explain the higher rate of open/close surgery in the upfront group.

Lastly, our cohort does include patients with short 30-min oxaliplatin HIPEC. As this HIPEC regimen is currently deemed ineffective, it could possibly affect the outcomes in our cohort. However, only 20 patients out of the 98 patients receiving preoperative chemotherapy received oxaliplatin HIPEC at subsequent CRS. Furthermore, it is currently not proven that any HIPEC regimen is effective in this setting (pending several trials). While it is a noteworthy limitation, the main driver of efficacy in this study is not the HIPEC regimen, but the conversion to CRS, which is increased with the use of targeted therapy in conjunction with doublet chemotherapy.

### Strengths

The strength of this study lies in its real-world approach, which involves capturing all patients at the initial MDT decision stage and comprehensively tracking outcomes across various systemic treatment strategies. Unlike prior studies that focused exclusively on cohorts undergoing successful CRS, this study mitigated selection bias by including patients who were not selected for surgery. This inclusivity enables broader evaluation of treatment efficacy.

### Future directions and conclusion

Further randomized controlled trials, such as the ongoing CAIRO6 study, where bevacizumab was administered preoperatively, are essential to validate these findings [13], 14]. Notably, the CAIRO6 study requires upfront resectability because patients are randomized in the other arm to upfront surgery which differs from our study. Nonetheless, the recent abstract on CAIRO6 reported that perioperative chemotherapy could not improve overall survival to the prespecified degree. Progression-free and disease-free survival did differ significantly between the arms, and subgroup analysis may still show relevant overall survival benefits in certain patients [20]. Despite this recent trial, the understanding and role of targeted therapy remains undefined and poorly investigated. To the best of our knowledge, our study is unique in evaluating preoperative therapy while maintaining patients not selected for surgery within the cohort. Additional studies are needed to assess targeted therapy on a larger scale in a prospective manner. In conclusion, targeted therapy improves overall survival in CRC-PM in patients that are not candidates for upfront CRS and HIPEC. Bevacizumab is associated with improvement over EGFR targeted treatment in a subgroup analysis. The combination of FOLFIRI and bevacizumab may be an optimal regimen to test in a future trial in this setting.
